# Pediatric lingual ectopic thyroid: two contrasting cases and a narrative literature review

**DOI:** 10.3389/fped.2026.1866654

**Published:** 2026-08-20

**Authors:** Zifeng Shi, Chengpin Tao

**Affiliations:** 1 Department of Radiology, Anhui Provincial Children’s Hospital, Hefei, Anhui, China; 2 Department of Pediatric Urology, Anhui Provincial Children’s Hospital, Hefei, Anhui, China

**Keywords:** base of tongue, children, ectopic thyroid, hypothyroidism, misdiagnosis

## Abstract

**Objective:**

To report two contrasting pediatric cases of lingual ectopic thyroid, one involving misdiagnosis and inadvertent excision and the other involving correct diagnosis and conservative treatment, and to summarize the diagnostic and therapeutic lessons through a narrative literature review.

**Methods:**

The clinical data of two children with ectopic thyroid were retrospectively reviewed. Case 1 was a 2-year- and 11-month-old girl with a mass at the base of the tongue that was misdiagnosed as a thyroglossal duct cyst and surgically excised. Case 2 was a 9-year-old girl who presented with a foreign-body sensation in the pharynx and was diagnosed with ectopic thyroid after systematic evaluation and treated conservatively. This report was designed as a two-case report/small case series with a narrative literature review. Relevant English-language literature on pediatric ectopic thyroid, lingual thyroid, radionuclide scintigraphy, conservative treatment, surgery, and radioiodine therapy was reviewed to support the discussion of diagnosis and management.

**Results:**

In Case 1, preoperative ultrasonography and computed tomography (CT) suggested a thyroglossal duct cyst. Thyroid function testing and radionuclide scintigraphy were not performed, and erroneous excision of the lesion resulted in overt hypothyroidism requiring long-term oral levothyroxine sodium replacement therapy. Radionuclide scintigraphy was not performed because nuclear medicine examination was unavailable at our institution. In Case 2, cervical ultrasonography, thyroid function testing, and contrast-enhanced CT confirmed ectopic thyroid at the base of the tongue with hypothyroidism. Radionuclide scintigraphy was also not performed because of the same institutional limitation. After oral levothyroxine sodium treatment, thyroid function returned to normal and symptoms were markedly relieved. In both patients, no normally located thyroid gland was identified in the neck, indicating ectopic thyroid without eutopic thyroid tissue.

**Conclusion:**

Ectopic thyroid should be considered before excision of pediatric tongue-base or anterior midline neck masses, especially when a normally located thyroid gland is not clearly identified. Thyroid function testing and cervical ultrasonography are appropriate initial evaluations. CT or MRI may be used when anatomical detail is required, when the diagnosis is uncertain, or when surgery is being considered. Radionuclide scintigraphy should be considered to confirm functional thyroid tissue and determine whether the ectopic thyroid represents the only functioning thyroid tissue. Treatment should be individualized, with priority given to preservation of thyroid function.

## 1 Introduction

Ectopic thyroid is a rare embryologic developmental anomaly caused by abnormal migration of the thyroid primordium during embryogenesis (1, 2). It is uncommon in clinical practice and is particularly likely to be overlooked or misdiagnosed in children because of its nonspecific manifestations. If ectopic thyroid is not recognized preoperatively and is mistakenly excised as another mass at the base of the tongue or as an anterior midline neck lesion, the only functional thyroid tissue may be lost, leading to permanent hypothyroidism and the need for lifelong thyroid hormone replacement therapy. In children, who are in a critical stage of growth and development, such an outcome may have important effects on physical growth, neurodevelopment, and quality of life, and may also impose a substantial burden on the family. Herein, we report two pediatric cases of ectopic thyroid at the base of the tongue: one case in which misdiagnosis and erroneous excision led to serious consequences, and one case in which standardized diagnosis and treatment resulted in a favorable outcome. A review of the relevant literature is also provided to improve clinicians’ understanding of this condition, strengthen awareness of differential diagnosis, standardize diagnostic and therapeutic procedures, and reduce misdiagnosis and inappropriate treatment.

## 2 Methods/narrative literature review

This study was designed as a two-case report/small case series with a narrative literature review. The clinical data, imaging findings, laboratory results, treatment strategies, and follow-up outcomes of two pediatric patients with lingual ectopic thyroid were retrospectively reviewed.

A narrative literature search was performed in PubMed, Web of Science, and Embase using the terms “ectopic thyroid,” “lingual thyroid,” “children,” “pediatric,” “thyroglossal duct cyst,” “hypothyroidism,” “ultrasonography,” “computed tomography,” “magnetic resonance imaging,” “radionuclide scintigraphy,” “99mTc-pertechnetate,” “SPECT/CT,” “levothyroxine,” “surgery,” and “radioiodine therapy.” Relevant English-language articles concerning the diagnosis and management of pediatric ectopic thyroid were reviewed. Because this was not a systematic review, formal study selection, quality assessment, evidence grading, and meta-analysis were not performed.

## 3 Clinical data

### 3.1 Case 1

**Figure 1:**
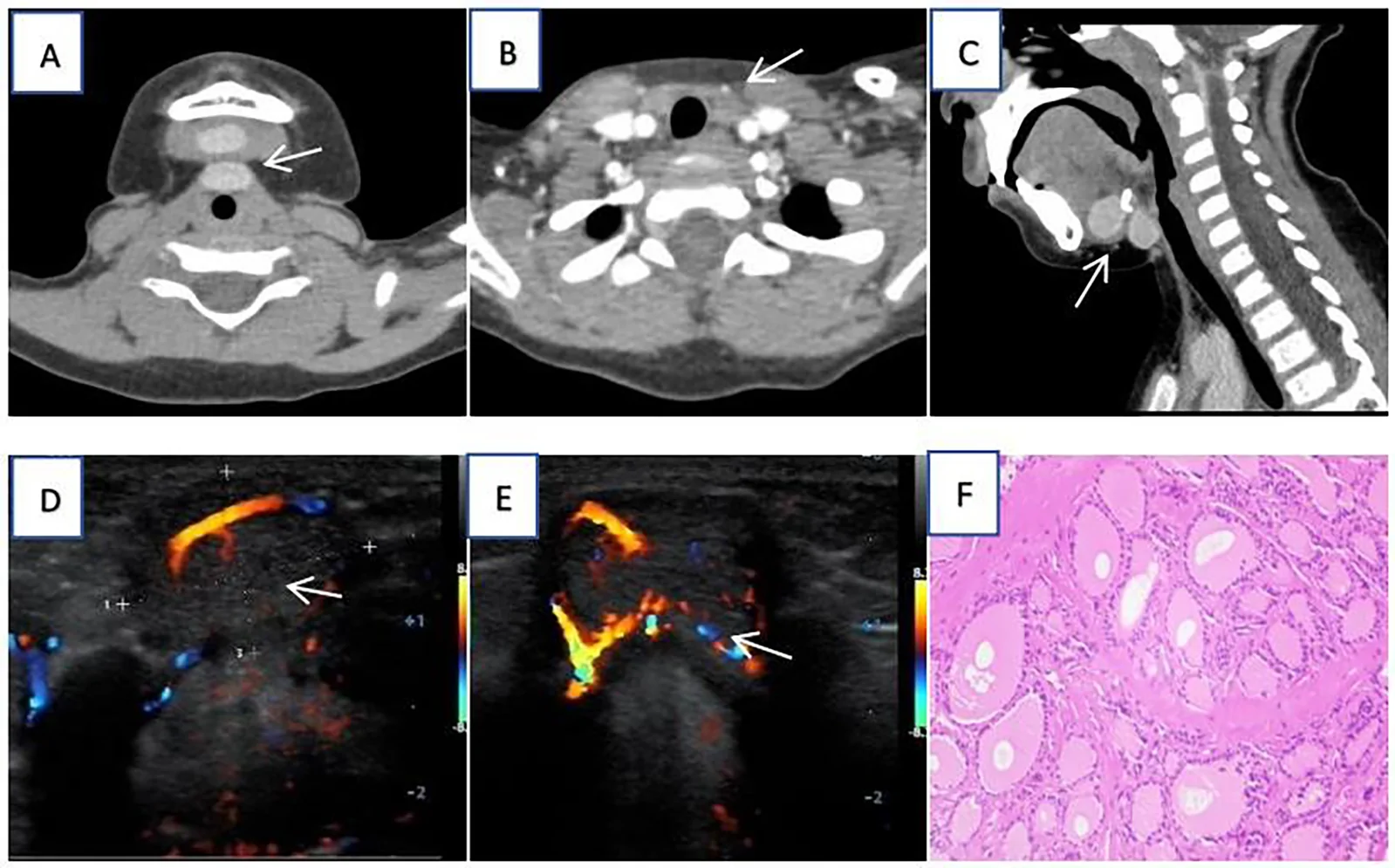
Preoperative imaging and postoperative pathologic findings of case 1. **(A)** Tongue-base mass later confirmed as ectopic thyroid tissue (white arrow). **(B)** No definite gland was observed in the normal thyroid bed; low-density enhanced tissue was misinterpreted as normally located thyroid tissue (white arrow). **(C)** The tongue-base nodule showed no obvious enhancement and was initially misdiagnosed as a thyroglossal duct cyst. **(D,E)** Color Doppler ultrasonography showed peripheral blood-flow signals in the lesion (white arrows). Although the lesion was initially interpreted as a thyroglossal duct cyst, the solid nature of the mass, blood-flow signals, and absence of a clearly demonstrated eutopic thyroid gland retrospectively supported the diagnosis of lingual ectopic thyroid. **(F)** Postoperative pathology (hematoxylin and eosin staining,   × 100) showed thyroid tissue.

A 2-year- and 11-month-old girl presented with a mass at the base of the tongue that had been noticed for more than 1 month. She had no obvious dysphagia, dyspnea, hoarseness, fever, or cough. Her past medical and family histories were unremarkable. Specialist examination showed a pink, round mass at the base of the tongue with a smooth surface, a diameter of approximately 1.5 cm, a firm consistency, and no obvious tenderness. No obvious abnormality was palpable in the neck. Electronic laryngoscopy showed no definite abnormality. Subsequent cervical ultrasonography and CT both suggested a thyroglossal duct cyst. Although cervical ultrasonography was performed, the examination mainly focused on the tongue-base lesion, and the absence of normally located thyroid tissue in the thyroid bed was not adequately recognized before surgery. Radionuclide scintigraphy was not performed because nuclear medicine examination was unavailable at our institution. At that time, the lesion was considered a thyroglossal duct cyst, and referral for functional thyroid imaging was not arranged before surgery. The patient subsequently underwent excision of the tongue-base mass under general anesthesia. Intraoperatively, the mass had an intact capsule and was solid, and it was completely excised. Postoperative pathologic examination showed thyroid tissue from the base of the tongue. The preoperative imaging and postoperative pathologic findings are shown in Figure 1. One week after surgery, the child developed sluggish responses, somnolence, loss of appetite, constipation, and dry skin. Repeat thyroid function testing showed thyroid-stimulating hormone (TSH) > 10.00 mIU/L, free thyroxine (FT4) 2.13 pmol/L, and total triiodothyronine (TT3) 0.61 nmol/L. Cervical ultrasonography showed no thyroid tissue in the normal thyroid bed. Based on the pathologic and imaging findings, postoperative iatrogenic hypothyroidism was diagnosed. Oral levothyroxine sodium replacement therapy was initiated at 25 μg/day, and the dose was subsequently adjusted according to thyroid function. Because the patient's body weight at treatment initiation was not completely documented in the available retrospective record, the levothyroxine dose could not be reliably expressed as μg/kg/day. During 2 years of follow-up, thyroid function was maintained within the normal range; however, long-term medication was required, and growth and development still require continuous monitoring.

### 3.2 Case 2

**Figure 2:**
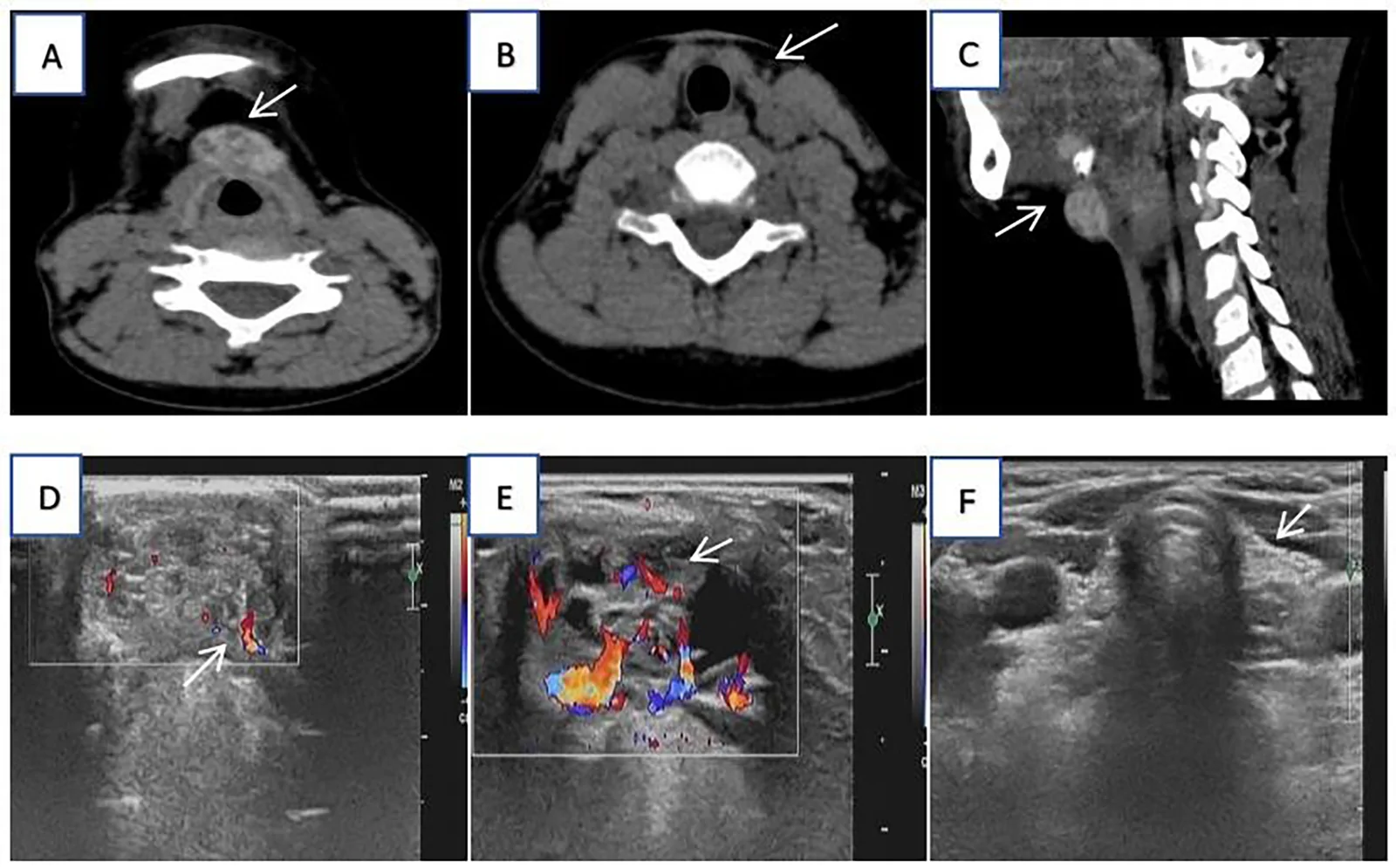
Imaging findings of case 2. **(A)** Ectopic thyroid tissue adjacent to the hyoid bone (white arrow). **(B)** No gland was visualized in the normal thyroid bed (white arrow). **(C)** Nodular ectopic thyroid tissue was observed above and below the hyoid bone. **(D,E)** Color Doppler ultrasonography showed blood-flow signals within the lesion (white arrows), supporting the diagnosis of ectopic thyroid. **(F)** No thyroid tissue was visualized in the normal paratracheal thyroid bed. The absence of eutopic thyroid tissue, together with hypothyroidism and vascularized tongue-base nodules, supported the diagnosis of lingual ectopic thyroid without normally located thyroid tissue.

A 9-year-old girl presented with a foreign-body sensation in the pharynx accompanied by mild swallowing discomfort for 2 weeks. She had no obvious dyspnea or hoarseness. Occasional choking was noted during eating, but there was no fever or cough. She had been previously healthy, and there was no family history of thyroid disease. Specialist examination revealed a round, elevated midline mass at the base of the tongue, with a smooth surface, measuring approximately 1.8 cm × 1.5 cm, firm in consistency, and without obvious tenderness. No thyroid tissue was palpable in the neck. Thyroid function testing showed TSH 8.56 mIU/L, FT4 8.12 pmol/L, and TT3 1.15 nmol/L, indicating hypothyroidism. Cervical ultrasonography showed no definite thyroid gland in the normal thyroid bed. Two nodular hypoechoic lesions with clear margins were observed at the base of the tongue; the larger lesion measured approximately 1.9 cm × 1.7 cm × 1.6 cm and showed a small amount of internal blood-flow signal. Ectopic thyroid was considered. Contrast-enhanced CT showed two quasi-round hyperdense nodular lesions at the base of the tongue; the larger lesion measured approximately 1.8 cm × 1.7 cm × 1.6 cm and showed mild heterogeneous enhancement after contrast administration. No thyroid tissue was visualized in the normal thyroid bed. The imaging findings are shown in Figure 2. Based on the clinical manifestations, laboratory findings, and imaging results, ectopic thyroid at the base of the tongue without eutopic thyroid tissue, complicated by hypothyroidism, was diagnosed. Radionuclide scintigraphy was not performed because nuclear medicine examination was unavailable at our institution. The diagnosis was established based on thyroid function testing, cervical ultrasonography, and contrast-enhanced CT, which showed ectopic thyroid tissue at the base of the tongue and absence of thyroid tissue in the normal thyroid bed.Because the child had no obvious compressive symptoms and the ectopic thyroid represented the only functional thyroid tissue, oral levothyroxine sodium was administered at 25 μg/day. Because the body weight at treatment initiation was not completely documented in the retrospective record, the dose could not be reliably expressed as μg/kg/day.One month after treatment, thyroid function had returned to normal, and the foreign-body sensation in the pharynx was markedly relieved. Although the lesion appeared slightly reduced clinically, quantitative follow-up measurements were not available because follow-up imaging was not routinely performed. During 6 months of follow-up, the patient's condition remained stable, thyroid function was maintained within the normal range, and growth and development were appropriate for age.The clinical features, diagnostic evaluation, treatment, and outcomes of the two cases are summarized in Table 1.

**Table 1:** Comparison of the clinical features, diagnostic evaluation, treatment, and outcomes of the two cases.

Variable	Case 1	Case 2
Age/Sex	2 years 11 months/Female	9 years/Female
Main presentation	Tongue-base mass noticed for more than 1 month	Foreign-body sensation in the pharynx with mild swallowing discomfort
Symptoms of compression	No obvious dysphagia or dyspnea before surgery	Mild swallowing discomfort and occasional choking; no obvious dyspnea
Preoperative thyroid function	Not tested	TSH 8.56 mIU/L, FT4 8.12 pmol/L, TT3 1.15 nmol/L
Ultrasonography	Lesion misdiagnosed as thyroglossal duct cyst; normal thyroid bed was not adequately evaluated	No definite thyroid gland in the normal thyroid bed; tongue-base hypoechoic nodules with blood-flow signals
CT/MRI findings	CT suggested thyroglossal duct cyst; cervical muscle was misinterpreted as thyroid tissue	Contrast-enhanced CT showed hyperdense nodular lesions at the base of the tongue; no thyroid tissue in the normal thyroid bed
Radionuclide scintigraphy	Not performed because nuclear medicine examination was unavailable at our institution	Not performed because nuclear medicine examination was unavailable at our institution
Eutopic thyroid tissue	No normally located thyroid tissue identified after surgery	No normally located thyroid tissue identified
Final diagnosis	Lingual ectopic thyroid without eutopic thyroid tissue; postoperative iatrogenic hypothyroidism	Lingual ectopic thyroid without eutopic thyroid tissue with hypothyroidism
Treatment	Surgical excision followed by levothyroxine replacement therapy	Conservative levothyroxine therapy
Outcome	Thyroid function controlled with long-term replacement therapy	Symptoms relieved and thyroid function normalized
Follow-up duration	2 years	6 months

## 4 Discussion

### 4.1 Embryologic basis and classification of ectopic thyroid

Ectopic thyroid is a rare embryologic developmental anomaly, with an estimated incidence of 0.3 to 1.0 per 100,000 in the general population and accounting for 0.01% to 0.03% of all thyroid diseases. It occurs more frequently in females, who account for approximately 65% to 80% of cases (3, 4). Several mechanisms have been proposed, but most investigators believe that ectopic thyroid results from insufficient or excessive descent of the thyroid primordium during embryonic development. Therefore, it most commonly occurs in the midline region extending from the base of the tongue to the mediastinum. According to the developmental status of the thyroid gland, ectopic thyroid can be classified as ectopic thyroid without eutopic thyroid tissue and accessory ectopic thyroid (5). In the former, no normally located thyroid tissue is present in the neck; this form accounts for approximately 70% of ectopic thyroid cases, and approximately 90% of such cases are located at the base of the tongue, where they are also known as lingual thyroid (6–9). Ectopic thyroid in other locations is extremely rare (10, 11). In accessory ectopic thyroid, ectopic thyroid tissue coexists with a normally located cervical thyroid gland. Adenoma and malignant transformation may also occur in ectopic thyroid tissue (12).

Many patients have no obvious clinical symptoms. In female patients, the ectopic gland may be discovered because of compensatory enlargement during puberty, pregnancy, or menopause. In childhood, ectopic thyroid is often more occult, and most cases involve lingual thyroid. Because early symptoms are often atypical, the condition is frequently detected only after the mass enlarges and compresses surrounding tissues, causing a foreign-body sensation in the pharynx, swallowing discomfort, snoring during sleep, or manifestations of hypothyroidism. Misdiagnosis is therefore common. Among the two children reported in this study, Case 2 presented with a foreign-body sensation in the pharynx and had relatively typical symptoms, whereas Case 1 had no obvious discomfort and was discovered only because of a mass, making the condition more likely to be overlooked.The contrasting clinical courses of these two patients highlight the importance of considering ectopic thyroid in the differential diagnosis of pediatric tongue-base or anterior midline neck masses before any excisional procedure is planned.

### 4.2 Standardized diagnostic workflow for pediatric ectopic thyroid

Based on the relevant literature and the present cases, the standardized diagnosis of pediatric ectopic thyroid should follow a systematic workflow. First, clinical screening is essential. For midline neck masses, especially masses at the base of the tongue, the normal thyroid region should be routinely examined to determine whether a gland can be palpated. If no definite thyroid tissue is palpable, ectopic thyroid without eutopic thyroid tissue should be highly suspected (9). A careful history and physical examination should also assess symptoms related to airway obstruction, dysphagia, sleep-disordered breathing, bleeding, infection, and hypothyroidism.

Second, laboratory testing is a fundamental component of the initial evaluation. Thyroid function testing, including TSH, free triiodothyronine (FT3), and FT4, helps determine whether the child has thyroid dysfunction. Patients with ectopic thyroid may have varying degrees of hypothyroidism (13, 14). In Case 1, preoperative thyroid function testing may have increased clinical suspicion regarding the nature of the lesion. Therefore, thyroid function testing should be performed before surgical treatment of a tongue-base or midline neck mass when ectopic thyroid is included in the differential diagnosis.

Third, imaging plays an important role in diagnosis. Ultrasonography is the preferred initial examination because it can determine whether a gland is present in the normal thyroid bed and can assess the location, size, margins, internal echogenicity, and blood supply of the mass (15). However, ultrasonography is operator-dependent, and careful evaluation of the normal thyroid bed is essential. In Case 1, ultrasonography focused mainly on the sublingual lesion, while systematic evaluation of the thyroid bed was insufficient, which contributed to failure to recognize the absence of eutopic thyroid tissue and resulted in preoperative misdiagnosis. CT and magnetic resonance imaging can demonstrate the relationship between the lesion and surrounding structures in multiple planes and are valuable for localization and treatment planning. Nevertheless, CT should not be recommended as a routine examination for all pediatric patients because of radiation exposure. CT or MRI should be considered when detailed anatomical information is needed, when the diagnosis remains uncertain, when the lesion is large or symptomatic, or when surgery is being considered. MRI may be preferred in selected children when radiation avoidance is particularly important and sufficient anatomical information can be obtained.

Radionuclide thyroid scintigraphy, including 99mTc-pertechnetate or radioiodine imaging, is a valuable nuclear medicine technique for confirming functional thyroid tissue and determining whether ectopic thyroid tissue represents the only functioning thyroid tissue (16). In particular, when cervical ultrasonography fails to identify a normally located thyroid gland or when surgery is being considered, scintigraphy or SPECT/CT can provide important functional and anatomical information. However, nuclear medicine examination was not available at our institution, and therefore radionuclide scintigraphy was not performed in either of the present cases. This was an important limitation, especially in Case 1, in which lack of preoperative functional imaging may have contributed to misdiagnosis and inadvertent excision. In institutions without nuclear medicine facilities, referral to a center with radionuclide imaging should be considered when ectopic thyroid is suspected, when no eutopic thyroid gland is identified, or when surgical treatment is being planned.

For patients in whom the diagnosis remains unclear after the above examinations or in whom malignancy is suspected, pathologic evaluation may be considered, including fine-needle aspiration cytology or intraoperative frozen-section examination. However, if ectopic thyroid without eutopic thyroid tissue is suspected, puncture should be performed cautiously to avoid hematoma, infection, or functional injury (17). Although intraoperative frozen-section examination may compensate for insufficient preoperative diagnosis, it should still be based on adequate preoperative assessment.Most importantly, excision should not be performed before confirming whether the lesion represents the only functional thyroid tissue.

### 4.3 Treatment principles for ectopic thyroid

Treatment of pediatric ectopic thyroid should be individualized, with the central aim of preserving thyroid function and avoiding unnecessary surgical excision. Because some ectopic thyroids, especially those without eutopic thyroid tissue, may represent the child's only functional thyroid tissue, treatment decisions must be made on the basis of sufficient preoperative evaluation.

For children without obvious symptoms, progressive enlargement, or compressive manifestations, regular follow-up observation may be adopted. Follow-up should include clinical symptoms, thyroid function, and imaging changes to dynamically assess disease progression. For children with hypothyroidism, levothyroxine sodium replacement therapy is the preferred treatment (18). This therapy not only corrects thyroid dysfunction but also suppresses TSH secretion, thereby reducing stimulation of ectopic thyroid tissue and, to some extent, relieving symptoms and controlling mass enlargement. In Case 2, thyroid function returned to normal and the foreign-body sensation in the pharynx was markedly relieved after levothyroxine sodium therapy. Because no normally located thyroid tissue was identified, long-term levothyroxine replacement is required, with periodic endocrine evaluation to adjust treatment according to thyroid function, growth, and developmental status. The dose of levothyroxine should be individualized according to age, body weight, thyroid function, symptoms, and growth status, with regular endocrine follow-up.

For patients with hypertrophic lingual ectopic thyroid causing obstructive symptoms, I-131 therapy has been reported as a potential nonsurgical treatment option in selected cases, particularly when surgery is contraindicated or declined. However, in children, I-131 therapy should be considered with great caution because of radiation exposure, possible loss of residual thyroid function, and the need for long-term endocrine follow-up. Such treatment should be reserved for carefully selected patients and performed in experienced nuclear medicine centers after multidisciplinary evaluation.

Recently, radiofrequency ablation (RFA) has emerged as a minimally invasive treatment option for selected benign thyroid lesions. However, its application in ectopic thyroid tissue remains rare, and evidence regarding its safety, efficacy, and long-term outcomes in children is insufficient. Therefore, RFA should currently be considered only in highly selected cases within experienced centers and after multidisciplinary evaluation.Surgical treatment should be performed only under strict indications, mainly including marked compressive symptoms affecting breathing or swallowing, recurrent bleeding or infection of the mass, suspected or confirmed malignant transformation, and persistent worsening of symptoms despite standardized conservative treatment (19). In children with confirmed normally located thyroid tissue, resection of the ectopic lesion may be considered after adequate evaluation. In children with ectopic thyroid without eutopic thyroid tissue, however, the ectopic tissue often represents the only functional thyroid tissue; therefore, surgery must be considered with particular caution, with full consideration of the risk of permanent postoperative hypothyroidism and lifelong replacement therapy (20). In Case 1, the only functional thyroid tissue was completely removed because of preoperative misdiagnosis, ultimately causing postoperative iatrogenic hypothyroidism. This provides a profound clinical lesson. Therefore, surgery for suspected pediatric ectopic thyroid should ideally be planned through multidisciplinary collaboration among otolaryngologists, pediatric surgeons, endocrinologists, radiologists, nuclear medicine physicians, and anesthesiologists, particularly when airway symptoms or operative treatment are involved.

### 4.4 Special considerations in pediatric patients

Children are in a critical stage of growth and development, and thyroid hormone is essential for central nervous system development and physical growth. If thyroid hormone deficiency is not corrected promptly, irreversible consequences such as intellectual disability, growth retardation, and learning impairment may occur (21). Therefore, in pediatric ectopic thyroid, it is particularly important to determine preoperatively whether the ectopic tissue is the only thyroid tissue. Once erroneously excised, the child will face lifelong replacement therapy, and precise dose adjustment, long-term adherence, and monitoring of growth and development will all present ongoing challenges (22).

In the present two cases, insufficient awareness of this condition contributed to diagnostic difficulty. In Case 1, both preoperative physical examination and contrast-enhanced CT misidentified cervical muscle as thyroid tissue, while ultrasonography focused only on the sublingual lesion and did not adequately evaluate the normal thyroid bed. These factors led to misdiagnosis, erroneous excision, and lifelong hypothyroidism. In contrast, Case 2 achieved a favorable outcome through standardized diagnosis and treatment. The diagnostic and therapeutic processes and outcomes of the two cases provide a striking contrast and an important lesson. Standardized preoperative evaluation and multidisciplinary collaboration can minimize misdiagnosis and inappropriate treatment.

This report also reflects a practical challenge in primary or non-specialized medical centers. When nuclear medicine imaging is unavailable, clinicians should not simply proceed to excision based on conventional imaging alone. Instead, the absence of a normal thyroid gland on cervical ultrasonography or CT/MRI should prompt further endocrine evaluation, careful reassessment of the diagnosis, and referral to a center capable of radionuclide scintigraphy when needed. This is especially important in children because unnecessary removal of the only functioning thyroid tissue may have lifelong consequences.

### 4.5 Limitations

This report has several limitations. First, only two pediatric cases were included, and the findings should therefore be interpreted cautiously. Second, the retrospective design limited the availability of some clinical details, including the exact preoperative thyroid function in Case 1 and body-weight-adjusted levothyroxine doses. Third, radionuclide thyroid scintigraphy was not performed because nuclear medicine examination was unavailable at our institution, which limited functional confirmation of ectopic thyroid tissue, particularly in Case 1. Therefore, referral to centers with nuclear medicine facilities should be considered when ectopic thyroid is suspected, no eutopic thyroid gland is identified, or surgery is being planned. Finally, the follow-up duration differed between the two cases, and the follow-up period in Case 2 was relatively short. Despite these limitations, the two cases provide a clinically meaningful contrast between misdiagnosis with inadvertent excision and standardized conservative management, although broader recommendations should be interpreted cautiously because of the small sample size.

## 5 Conclusion

In conclusion, these two pediatric cases illustrate an important diagnostic pitfall in the evaluation of tongue-base and anterior midline neck masses. Ectopic thyroid should be considered before excision, particularly when normally located thyroid tissue is not clearly identified. Thyroid function testing and cervical ultrasonography are appropriate initial evaluations, while CT or MRI may be useful when further anatomical assessment is required, when the diagnosis remains uncertain, or when surgery is being considered. Radionuclide scintigraphy should be considered when functional confirmation is needed or when surgery is being planned. Treatment should be individualized, with priority given to preserving thyroid function. Conservative management, including thyroid hormone replacement and careful surveillance, remains the preferred strategy in children when ectopic thyroid represents the only functioning thyroid tissue, whereas invasive treatments should be reserved for carefully selected patients. Because this report includes only two cases, these findings should be interpreted as clinical lessons rather than broad management recommendations.

## Data Availability

The anonymized data used and/or analyzed during the current study are available from the corresponding author upon reasonable request, subject to institutional privacy and ethical restrictions.
